# Lateral dorsal infundibular approach: an alternative option for the safe completion of difficult laparoscopic cholecystectomy

**DOI:** 10.1186/s12893-022-01894-4

**Published:** 2022-12-25

**Authors:** Juxian Song, Jian Chen, Shuguo Zheng

**Affiliations:** 1 Department of Hepatobiliary Surgery, The 925Th Hospital of the Chinese People’s Liberation Army, Guiyang, 550009 China; 2 Institute of Hepatobiliary Surgery, First Affiliated Hospital, Army Military Medical University, Shapingba District, Gaotanyan Main Street 29, Chongqing, 400038 China

**Keywords:** Cholelithiasis, Gallstones, Cholecystectomy, Laparoscopy, Surgical Procedures

## Abstract

**Background:**

Difficult laparoscopic cholecystectomy (LC) due to acute cholecystitis (AC) increases the risk of bile duct injuries and postoperative complications. Here, we added the lateral dorsal infundibular approach as an initial surgical maneuver during LC to improve outcomes.

**Methods:**

We describe the detailed technical procedure of the lateral dorsal infundibular approach in patients with AC resulting in difficult LC. This technique was developed after nearly 10 years of experience in laparoscopic surgery, and has been routinely used in the past 5 years. We also retrospectively analyzed the perioperative data for 469 patients with difficult LC.

**Results:**

A total of 469 patients with AC received difficult LC between July 2016 and June 2021, of which 438 (93.4%) performed a lateral dorsal infundibular approach. Sixty-four patients (13.6%) had variations of the hepatic bile duct and cystic duct according to preoperative magnetic resonance cholangiopancreatography, 438 patients (93.4%) received elective surgery, 31 (6.6%) received emergency surgery, and 10 (2.1%) underwent conversion. There was no postoperative bile leaks and no bile duct injuries in the described technique.

**Conclusion:**

During difficult LC, the critical view of safety can be gradually achieved by changing the surgical approach to achieve cholecystectomy.

## Background

Changes in the sociodemographic structure have led to a gradual increase in the incidence of acute cholecystitis (AC), due to the occurrence of gallstone disease among the aging population. Previous studies have shown that increasing age is significantly related to increases in surgical complications and perioperative mortality [1]. Surgeons will therefore face more severe challenges and should pay careful attention to the choice of surgical methods for AC.

Laparoscopic cholecystectomy (LC) has been widely recognized as a safe and effective treatment for gallstones. Its faster recovery, less pain, and shorter hospital stay compared with open surgery mean that LC has been the standard surgical treatment [2]. The initial limitations of LC such as poor haptic feedback and insufficient visibility meant that AC was a relative contraindication for LC, according to the guidelines. However, advancements in laparoscopic techniques and concepts, increasingly updated equipment, and improved understanding of the anatomy have expanded the surgical indications of LC. The 2018 Tokyo Guidelines has been revised to read “If both the patient and the facilities where the surgery is to be performed meet strict conditions, LC may also be performed as a straightforward procedure under certain conditions of Grade III cases” [3].

AC is caused by excessive gallbladder (GB) enlargement, edema, and congestion, making the Calot’s triangle relationship unclear. It is difficult to establish a “critical view of safety” (CVS) during LC, and the chances of bile duct injuries (BDI) and bleeding are increased, making it difficult to complete LC successfully. Conversion to laparotomy is then usually recommended as an important and indispensable bail-out procedure for difficult LC [4]. However, whether the occurrence of BDI is reduced after conversion remains unclear [5]. Nevertheless, techniques to address the issues of difficult LC have continued to be explored, and clinical observations of techniques such as laparoscopic subtotal cholecystectomy (STC) [6], “fundus-first” technique, “middle-first” approach, and posterior infundibular dissection have all demonstrated that surgery can be safe and effective [7]. Except for biliary repair, laparoscopic surgery can be used for most radical surgeries, including GB cancer [8].

Here, we report the technical details of the lateral dorsal infundibular approach, developed over the past 10 years, which has demonstrated success during difficult LC for the treatment of severe inflammation or gangrenous cholecystitis. The main aim of the procedure is to avoid BDI by using laparoscopic STC technique to explore and mark the infundibulum to determine the initial anatomical landmarks, from the lateral submucosal approach to the dorsal, until complete circumferential dissection of the infundibulum allows CVS, and the GB can then be removed in sections. We evaluated this technique in 469 cases of difficult LC.

## Methods

This study was approved by the Ethics Committee of the 925th Hospital of the Chinese People’s Liberation Army. In this single-center study, we retrospectively analyzed the medical records of 469 patients with pathologically confirmed AC who underwent difficult LC between July 2016 and June 2021. These patient records were reviewed by two senior surgeons, and only patients who met the Tokyo Guidelines 2018 criteria for the diagnosis of AC and performed the lateral dorsal infundibular approach were included in the analysis. Patients with gallbladder cancer confirmed by postoperative pathology were excluded. The data collected included the patients’ demographic characteristics (sex, age, postoperative pathology), and preoperative ultrasound and magnetic resonance cholangiopancreatography (MRCP) results. Perioperative data included the timing of surgery (elective/emergency), conversion, infundibulum management (fenestrating or reconstituting), bile leaks, retained stones, operation time, blood loss, hospitalization duration, and follow-up. The follow-up period ended in November 2021. Statistical analysis of the data was performed by SPSS 25.0 software, all data was analyzed by descriptive statistics.

## Technique

### Surgical visibility and decision-making

**Fig. 1:**
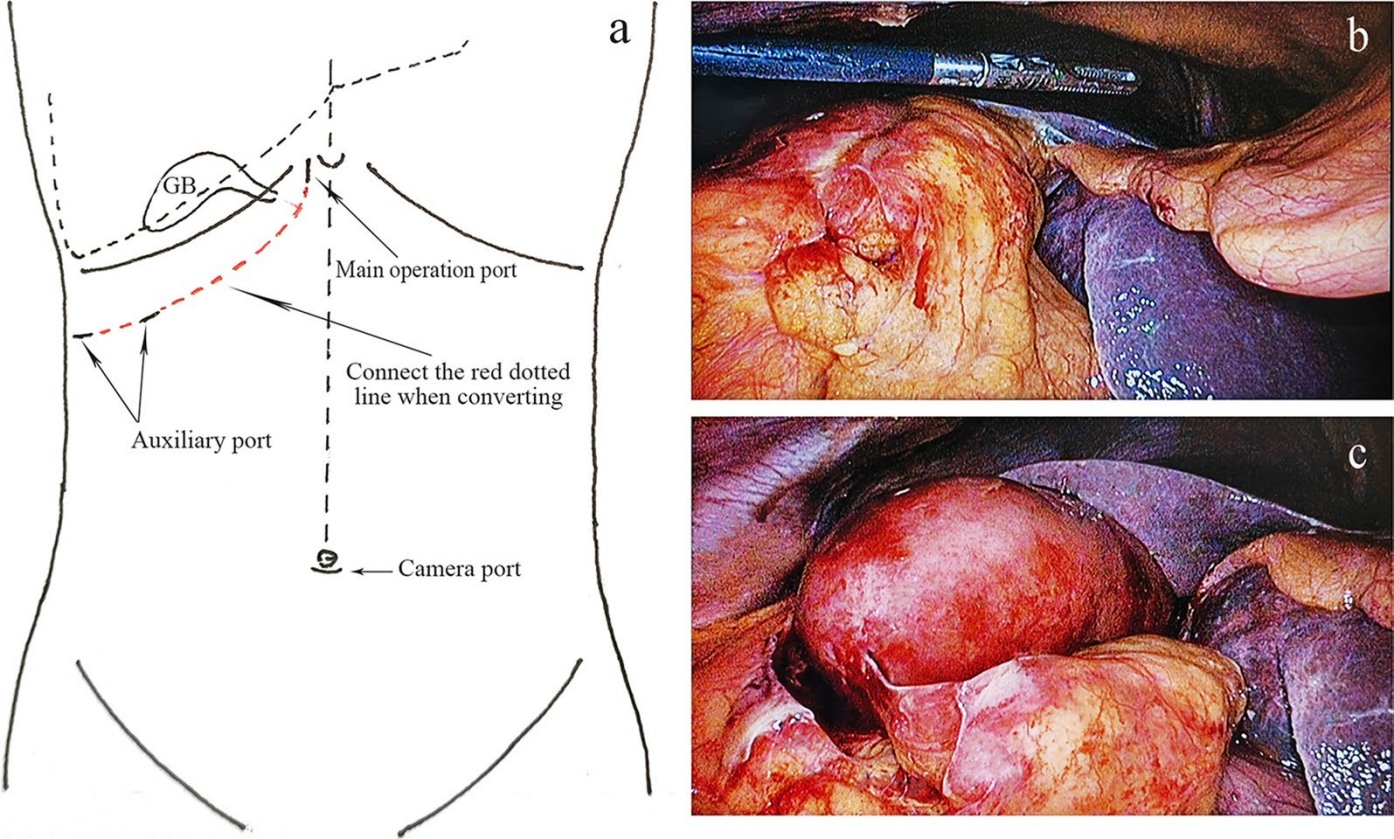
**a** Trocar placement. **b**, **c** Gallbladder adhesiolysis

The camera port position is located close to the bottom of the navel, in line with operating habits and taking account of the aesthetic results. According to the difficulty of operation, three or four ports were selected. The main operation port is a 10-mm trocar placed on the right side of the round hepatic ligament under the xiphoid process, and the other auxiliary ports are one or two 5-mm arc-shaped trocars placed under the ribs around the operating area (Fig. 1a). The first indication of severe GB inflammation or gangrene is usually encapsulated adhesions of the greater omentum, stomach, and transverse colon, resulting in no or partial exposure of the GB (Fig. 1b). If the adhesions along the serosal layer of the GB can be loosened using a suction device, ultrasonic scalpel, high-frequency surgical unit, or other instruments to gradually reveal the GB, Calot’s triangle, and common bile duct area, the procedure can be continued laparoscopically; otherwise conversion or intraoperative cholecystostomy drainage will be required. During this process, it is often necessary to cut the bottom of the GB to reduce pressure to facilitate grasping. At the same time, it is necessary to loosen the adhesions on the right side of the liver as much as possible to obtain a better visual field and facilitate the placement of postoperative drainage (Fig. 1c). After the release is complete, it is important to carefully distinguish the shape of the infundibulum and the trajectory of the common bile duct.

### Laparoscopic STC and infundibulum exploration

Adopting a middle-first approach [9] as the initial method involves dissection from the middle part of the GB, moving gradually towards the infundibulum after complete mobilization of the bottom and body. After cutting with an ultrasonic scalpel, the entire cavity is completely exposed, the bile is sucked out, and the stones are removed and placed temporarily in the abdominal cavity. The tissue at the cystic plate is preserved, and the body and bottom of the GB are removed along the boundary between the GB wall and the liver. The infundibulum is then checked and forceps are used to remove the incarcerated stones, and the excised tissue and stones are removed from the abdominal cavity together using the specimen-retrieval bag. The cystic plate mucosa is retained to reduce the bleeding caused by the plate/sheath system of the liver and to shorten the operation time, while the remaining tissue is destroyed using a high-frequency surgical unit. Bleeding during the resection process is treated with an ultrasonic scalpel and a high-frequency surgical unit. Because of the distance from Calot’s triangle and the liver, hemostasis is easy and effective.

**Fig. 2:**
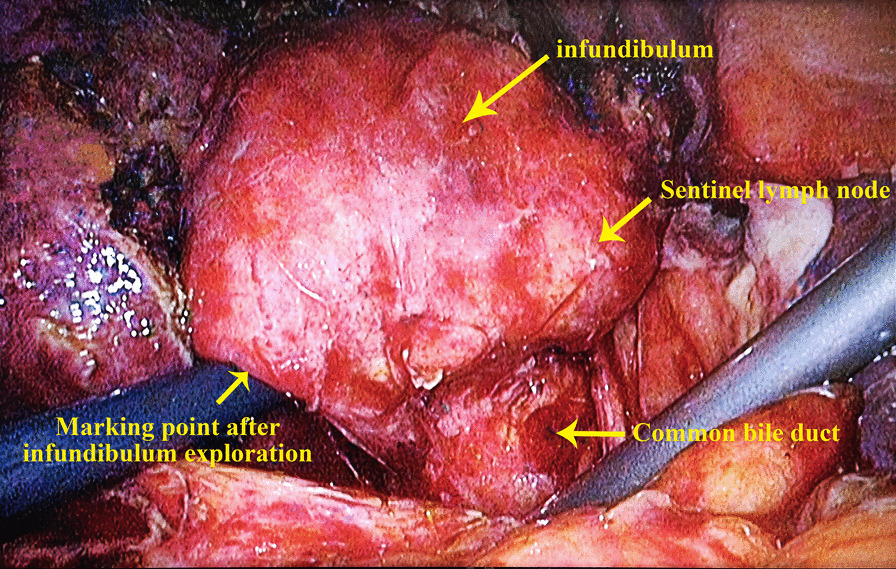
Exploration and marking of the infundibulum after subtotal cholecystectomy

After most of the GB has been removed and the infundibulum is completely exposed, the cavity can be further explored using endoscopic instruments or a choledochoscope. The aim is to avoid residual stones and determine if the cystic duct is completely closed. It is also important to explore the cavity if the anatomical landmarks are not obvious, and to evaluate and mark the boundaries of the infundibulum from the inside to the outside. Identification of the enlarged sentinel lymph node can also help to confirm the boundary, and is usually also the penetrating confluence point for subsequent dissection (Fig. 2).

### Posterior infundibular dissection

**Fig. 3:**
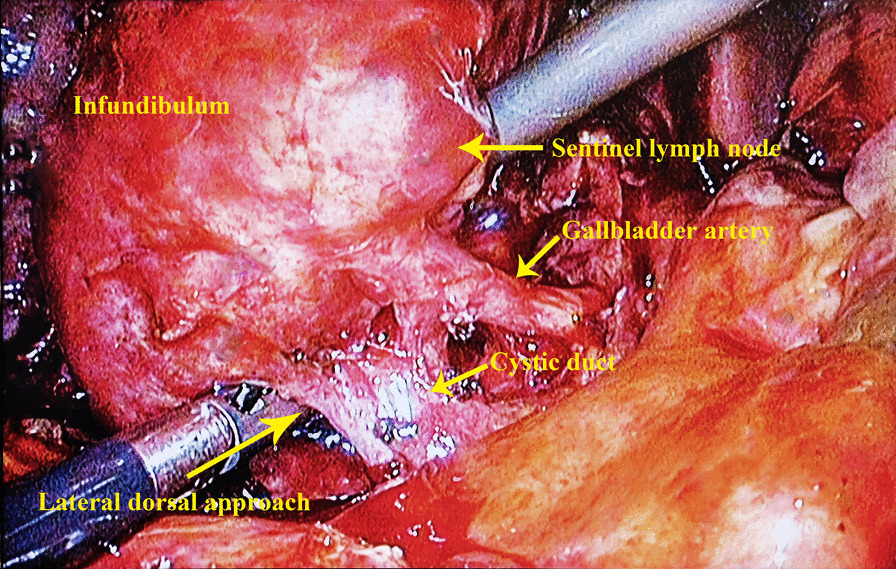
The lateral approach is dissected to the dorsal side along the junction of the serosal layer and the muscular layer until the cystic duct and arteries are completely exposed to achieve CVS

**Fig. 4:**
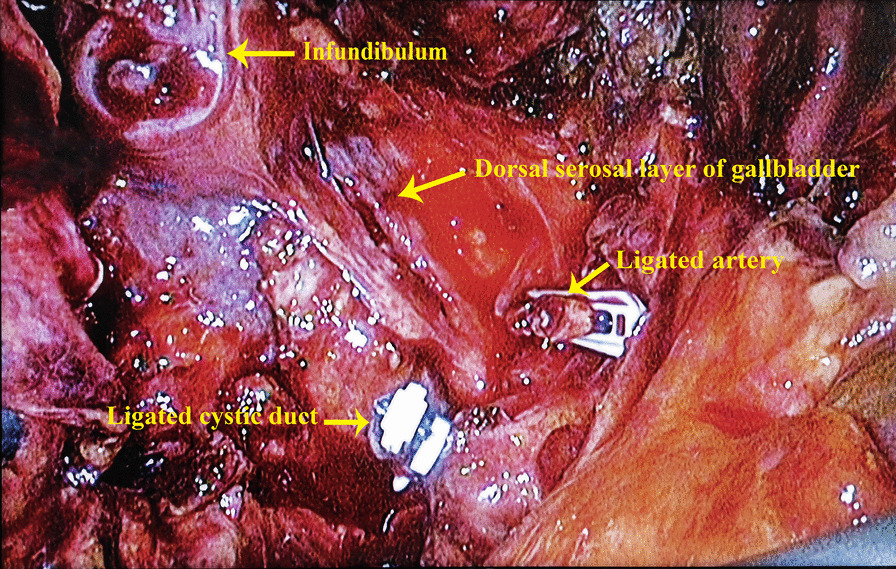
Ligation of the cystic duct and peeling off the infundibulum while retaining the serosal layer on the dorsal side

It is difficult to achieve CVS at this time, thus increasing the risk of injuring the bile ducts and blood vessels. We therefore chose the lateral infundibulum with a clear anatomical structure as the starting point, combined with the position of the right edge of the sentinel lymph node as the confluence of the dorsal circumferential dissection. The excessive edema and congestion of the tissue during acute inflammation makes the boundaries between the layers still obvious, and after opening the serosal layer, dissection can proceed dorsally along the boundary between the serosal layer and the muscular layer. While peeling off, the infundibulum should be pushed away from the distal end. During this process, it is necessary to identify the anterior and posterior branches of the GB artery until the entire shape is completely exposed and confirmed (Fig. 3). At this point, the dissection of the infundibulum is complete. To avoid BDI, the cystic duct can be dissected according to the actual situation. Once the CVS is reached, the confluence of the infundibulum and the cystic duct is ligated using Hem-o-lok or silk thread. Finally, the dorsal serosal layer is retained and the infundibulum is removed to achieve segmental resection (Fig. 4).

### Management of postoperative complications

Postoperative drainage is routine for observation of the condition. In most patients whose cystic duct is dissected and clipped, the duration of drainage is 3–7 days, and the indication for removal is no bile leaks confirmed by ultrasound. Patients who underwent fenestrating and reconstituting STC were discharged after the drainage was closed without bile leaks, and the drainage was removed in the outpatient clinic after ultrasound confirmation 1 week later. Postoperative bile leaks is a relatively common complication of LC, caused by postoperative regression of inflammation and edema and retraction of the sutures or recanalization of the cystic duct. If bile leaks does occur, treatment includes fasting, continuous abdominal drainage, total parenteral nutrition, and the timing of endoscopic retrograde cholangiopancreatography (ERCP) (including endoscopic sphincterotomy and temporary stent placement) according to the postoperative drainage volume. Following these treatments, bile leaks usually resolve within 1–2 weeks.

## Results

We retrospectively analyzed 469 cases of difficult LC. The patients included 176 men (37.5%) and 293 women (62.5%), aged 34–87 years (mean 55.1 years). The postoperative pathology showed AC in all cases, including 215 cases (45.8%) of gangrenous cholecystitis. All patients underwent preoperative ultrasound and MRCP. The thickness of the GB wall measured by ultrasound was 4–10 mm in 361 patients (77.0%) and > 10 mm in 108 patients (23.0%). Sixty-four patients (13.6%) had variations of the hepatic bile duct and cystic duct found by MRCP. Overall, 438 patients (93.4%) received elective surgery, 31 (6.6%) received emergency surgery, and 10 (2.1%) required conversion. The described technique was completed successfully in 434 patients (92.5%), 13 patients (2.8%) underwent fenestrating STC, and 22 (4.7%) underwent reconstituting STC. Postoperative bile leaks occurred in four patients (0.9%) (3 fenestrating, 1 reconstituting), of which three cases healed within 1–2 weeks after ERCP. The remaining patient who underwent reconstituting STC had bile leaks but refused ERCP treatment, and finally healed spontaneously after continuous drainage for 2 months. There were no retained stones after surgery. The mean operation time was 117.0 ± 34.5 min, the mean blood loss was 146.9 ± 53.2 ml, and the mean hospitalization duration was 7.6 ± 2.6 days. There were no injuries to blood vessels or bile ducts, no reoperation, no readmissions, and no deaths.

## Discussion

The rapid development of laparoscopic surgery has led to increasing reports of difficult LC worldwide. Consensus guidelines from the 2018 Boston Conference and the Tokyo Guidelines recommended standardized safety procedures, emphasizing the exposure of Rouviere’s sulcus and efforts to create CVS. If the CVS cannot be achieved due to scar formation or severe fibrosis during the operation, or if the Calot’s triangle is shrinking and the boundary is unclear, a bail-out approach should be considered, including conversion, STC, or the fundus-first technique. The common point of both STC and the fundus-first technique is the retention of the infundibulum. The advantages of the technique include no dissection of Calot’s triangle, reduced bleeding, shorter operation time, and avoidance of BDI. However, the disadvantages of both the fenestrating and reconstituting techniques include postoperative bile leaks [7]. Even long-term postoperative observation may reveal recurrent biliary events [10]. Bile leaks needs to be treated with continuous drainage or ERCP, thus increasing the length of hospital stay, delaying healing, and increasing the economic burden. Therefore, based on the surgeon’s laparoscopic experience and clinical observation, the advantages of laparoscopic local field magnification and fine dissection should be maximized to try to dissect and completely free the infundibulum and ligate it, to improve the postoperative outcomes.

The initial method of the infundibulum side boundary is used to reduce the dissection of Calot’s triangle and avoid the occurrence of BDI [11]. Our technique does not use a constant anatomical landmark as a reference point. After exploring the infundibulum, only the trajectory of the common bile duct needs to be identified on the anatomical plane, and the cystic duct and artery are clearly defined. The stripped plane can then be safely resected without other luminal structures. Maintaining the subserosal approach can effectively avoid the effects of anatomical variations such as hepatobiliary duct variation and abnormal cystic duct confluence, consistent with the international consensus principle of closely following the GB dissection to avoid BDI. Another important technical feature includes the preservation of the cystic plate, which is part of the plate/sheath system of the liver. Severe inflammation can cause tissue edema and vasodilation, making the boundary between the GB and liver unclear. The peeling process produces a large amount of smoke that reduces visibility during the operation. If the anatomical surface penetrates deep into the liver and damages the blood vessels, it will cause more bleeding, thus increasing the surgical trauma and operation time. We therefore chose the GB–liver junction with clear boundaries and easy hemostasis as the resection plane, and destroyed the remaining mucosa by heat, resulting in no cases of postoperative abdominal effusion.

BDI is the most serious complication of LC and presents a problem for hepatobiliary surgeons throughout their learning curve. The reported incidence of BDI was 0.23%, and although the incidence has declined in recent years, it remains higher following laparoscopic compared with open cholecystectomy [12]. BDI associated with LC ranges from minor injuries to complex hilar injuries, as classified by Strasberg et al., with the most severe type being type E injuries, including persistent stenosis, complete occlusion, resection, or bile duct division [13]. The occurrence of BDI not only increases the length of hospitalization and the economic burden, but also means that the patient is likely to face recurrent bile duct strictures, requiring secondary hepatectomy, biliary-enteric or hepato-enteric anastomosis, eventually leading to liver transplantation or death [14, 15]. BDI is also a common cause of medicolegal problems for surgeons [16]. Therefore, in the procedure of difficult LC, we make the following recommendations. (1) Severe inflammation and excessive compression of the swollen cysts will distort the local anatomical structure resulting in anatomical difficulties, and the complete removal of fat and fibrous tissues in Calot’s triangle cannot be achieved. In addition, dissection of the area should be minimized at this time to avoid BDI. (2) Detection of the infundibulum and identification of the sentinel lymph node allows the infundibulum to be dissected circumferentially after the lateral dorsal approach, while retaining the serosal layer of the dorsal part. (3) The limitation of the anatomical plane means that the trajectory of the common bile duct, junction of the infundibulum and the cystic duct, and the anterior and posterior branches of the artery entering the GB can be seen visually.

Preoperative examination is important to realize the above steps, including safe resection of the infundibulum, no BDI, and a low conversion rate. Detailed imaging examinations are critical for surgical planning, especially in cases of difficult GB assessments, such as patients with Mirizzi’s syndrome, aberrant hepatic ducts/cystic duct insertions, and rare cancers. Preoperative ultrasound can provide an initial impression, including diagnosis of gallstones, measurement of swelling, and GB wall thickness. However, we consider MRCP to be the most important and effective method for assessing the severity of cholecystitis inflammation and the extrahepatic bile ducts [17]. Magnetic resonance imaging (MRI) can show the degree of GB enlargement and the condition of common bile duct stones. Furthermore, the faster acquisition protocol allows for good tissue contrast and can also determine the condition of the GB wall, including thickness stratification, early edema, necrosis, or later fibrosis or scars. Importantly, MRCP can display the adjacency between the enlarged GB, the common hepatic duct, and the common bile duct, and can detect variations in the cystic duct and bile duct, providing the surgeon with a systematic understanding of the entire biliary system and GB before surgery, thus helping to avoid BDI. MRCP is important for the diagnosis, treatment planning, and prognosis of AC, and should be completed even before emergency surgery [18]. ERCP is used in cases where MRI examination is not possible and is necessary for the treatment of common bile duct stones. In addition, enhanced computed tomography can be used to rule out the possibility of tumors and to check the blood vessels in the hilar region [19]. Operative procedures in our institution rarely include intraoperative imaging techniques.

None of the patients in the current study had developed BDI by 5 years after difficult LC using the described technique. This method thus has several advantages (1) Surgical preservation of the cystic plate mucosa is simple and quick, saving operating time for subsequent procedures, and also reduces bleeding because the plate/sheath system of the liver is not penetrated. (2) Exploration of the infundibulum cavity not only avoids the occurrence of residual stones, but also helps to confirm the overall shape from the inside out, to lay the foundation for the next step of dissection. (3) The method can effectively avoid damage to blood vessels and hepatic bile ducts, and thus avoid BDI.

## Conclusions

Minimally invasive surgery has great benefits and is extremely attractive to patients and surgeons; however AC presents a challenge for surgeons carrying out LC. We believe that after completing the laparoscopic learning curve, surgeons can innovate and explore according to the patient’s actual condition, notably in the context of the rapid development of laparoscopic technologies and the integration and clinical application of various advanced technologies such as digital medicine and artificial intelligence. During difficult LC, CVS can be gradually achieved by changing the surgical approach and safely peeling off the infundibulum to achieve cholecystectomy.

## Data Availability

The datasets used and analysed during the current study are available from the corresponding author on reasonable request.

## Abbreviations

AC: Acute cholecystitis
LC: Laparoscopic cholecystectomy
GB: Gallbladder
CVS: Critical view of safety
BDI: Bile duct injuries
STC: Laparoscopic subtotal cholecystectomy
MRCP: Magnetic resonance cholangiopancreatography
ERCP: Endoscopic retrograde cholangiopancreatography
